# Characterization of a 0.5 × 0.5 mm plastic scintillating detector for small field dosimetry: Its performance and comparison with other commercially available plastic scintillating detectors

**DOI:** 10.1002/acm2.70205

**Published:** 2025-08-21

**Authors:** Milad Baradaran‐Ghahfarokhi, Beshoi Grees, Annie Cooney, Erica Northern, George Ding, Guozhen Luo, Kenneth Homann

**Affiliations:** ^1^ Department of Radiation Oncology Vanderbilt University Medical Center Nashville Tennessee USA

**Keywords:** detector correction factors, field output factors, plastic scintillating detector, small field dosimetry

## Abstract

**Background:**

Lack of an ideal detector for small field dosimetry has led to the development of many new types of detectors. Recent studies have shown that plastic scintillation detectors (PSDs) provide favorable dosimetric characteristics, such as minimal volume averaging and fluence perturbation effects, real time response rates, high signal to noise ratio (SNR), and independence to temperature, energy spectrum, dose rate, and irradiation direction, in the field of small field dosimetry, largely due to their small size and water‐equivalent composition materials, which eliminates the need for certain correction factors.

**Purpose:**

The goal of this study was to evaluate a new 0.5 × 0.5 mm^2^ PSD (called in this study Medscint0.5), with detailed uncertainty analysis, for its suitability in small field dosimetry and in comparison, to other commercially available PSDs.

**Methods:**

This detector characterization was performed for small field output factors, pre‐ and post‐ irradiation leakage, short‐term repeatability, angular dependency, dose rate and energy dependency, dose response linearity, and temperate dependency. The small field output factors measured with this detector were also evaluated to determine if any correction factors were needed for extremely small fields.

**Results:**

The Medscint0.5 showed negligible leakage (< 0.1%) and short‐term repeatability (< 0.1% response deviations), similar to other commercially available PSDs. It also demonstrated comparable dose rate (< 0.47%) and dose response linearity (< 0.73%) to an ion chamber. Moreover, it also showed excellent angular (< 0.30%) and temperature independence (< 0.38%) relative to other commercially available PSDs. Stereotactic cone factor measurements showed good agreement (< 1.10%) with MC for all SRS cone sizes for both 6 and 10 MV FFF beams. The total uncertainty for absolute dosimetry using this detector was found to be ∼1.1% at one standard deviation from the mean. The uncertainty can be reduced to 0.8% at one standard deviation when this detector is used for relative small field dosimetry, that is, field output measurements.

**Conclusion:**

The latest small size Medscint0.5 detector is suitable for small field dosimetry with its unique advantage in measuring field output factors without need of any correction factor even in measuring small circular fields down to 4 mm diameter.

## 1 INTRODUCTION

For small field dosimetry, the degree of lateral charged particle equilibrium and output factors decreases dramatically, which necessitates selection of a detector with appropriate size, density, atomic composition, and construction shape.^1^, ^2^ An ideal small field dosimeter displays certain preferred characteristics, including but not limited to, minimal volume averaging and fluence perturbation effects, real time response rates, high signal to noise ratio (SNR), and independence to temperature, energy spectrum, dose rate, and irradiation direction.^3^, ^4^, ^5^, ^6^, ^7^

Recent studies have shown that plastic scintillation detectors (PSDs) provide these favorable dosimetric characteristics in the field of small field dosimetry mainly because of their small size and water‐equivalent composition materials, which eliminates the need for certain correction factors for this type of detector.^4^, ^8^, ^9^, ^10^, ^11^ However, PSDs need extra signal processing techniques to subtract Cerenkov light, considered as noise, produced in optical transmission fibers, which can compromise the accuracy of dose measurements.^12^, ^13^, ^14^ Moreover, care should be taken during calibration of PSDs to account for differences across the beam's energy spectrum, which increases dosimetric uncertainty for small fields.^10^, ^11^

Using Monte Carlo (MC) simulations, Francescon et al. have shown that the Exradin W1 (Standard Imaging, Madison, Wisconsin, USA) PSDs required < 0.5% correction for all studied CyberKnife field sizes (down to 5 mm). Given their estimated total uncertainty of < 0.8% (k = 1),^15^, ^16^ they recommended using no corrections for scintillation detectors for small field output factor measurements. Morin et al. have performed a comparative study between cylindrical scintillating fiber detectors (multicladding SCSF‐78 M, Kuraray Co., Ltd., Tokyo, Japan), with 0.5 mm diameter and 1.0 mm length, and various other stereotactic dosimeters.^5^ Their results showed negligible volume‐averaging effect (∼1%) compared to silicon diodes, when PSDs diameters were placed perpendicular to the radiation beam.^5^ However, according to their findings, an accurate spectral discrimination method should be applied to subtract unwanted Cerenkov light from the signal. More recently, Das et al. characterized a new PSD system (Blue Physics, Tampa, Florida, USA) for small photon beams from a Varian TrueBeam linear accelerator (linac) (Varian Medical Systems, Palo Alto, California, USA).^4^ They stated that the studied PSD is a robust, reliable, and accurate detector that can be used for small field dosimetry with no correction factor. Nonetheless, they indicated that the size and shape of the PSDs can affect dosimetry in small fields due to the increased likelihood of photon scatter.^4^ Timakova et al. have evaluated dosimetric characteristics of the HYPERSCINT RP‐200 (HS RP‐200) PSD (Medscint Inc. Quebec, Quebec, Canada) system.^17^ The system is coupled with a 0.8 mm^3^ probe, diameter of 1 mm and length of 1 mm (labeled as Medscint1.0), and can subtract noise and optimize true signal isolation from the PSD to ensure accurate dose measurements. Their findings showed excellent dosimetric properties of this Medscint PSD system for small field dosimetry down to field sizes of 0.5×0.5 cm^2^.^17^ More recently, the manufacturers have produced a smaller probe size of 0.5 mm in diameter and 0.5 mm in length (Medscint0.5), which enables the acquisition of data with higher spatial resolution, especially for very small field sizes down to a 4 mm Cone. In addition to the smaller detector size, our measurements also utilized more advanced signal processing and spectral binning, and an upgraded five‐component calibration.

The latest, smaller Medscint0.5 probe has not been evaluated for small field dosimetry. In this study, a comprehensive detector characterization was performed for small field output factors, pre‐ and post‐ irradiation leakage, short‐term repeatability, angular dependency, dose rate and energy dependency, dose response linearity, and temperate dependency. Furthermore, an uncertainty analysis of the measurements using Medscint0.5 is included in this study.

Dosimetric characteristics of this detector were compared with other commercially available plastic scintillating detectors, namely, Blue Physics (Blue Physics, Tampa, Florida, USA) and Exradin W2 (Standard Imaging, Madison, Wisconsin, USA). Results of the output factor measurements were also compared with Monte Carlo (MC) simulations, using EGSnrc BEAMnrc (Version. 4‐r2.3.1, National Research Council, Canada), and PTW microSilicon diode (PTW, Freiburg, Germany) measurements.

## 2 MATERIALS AND METHODS

**FIGURE 1:**
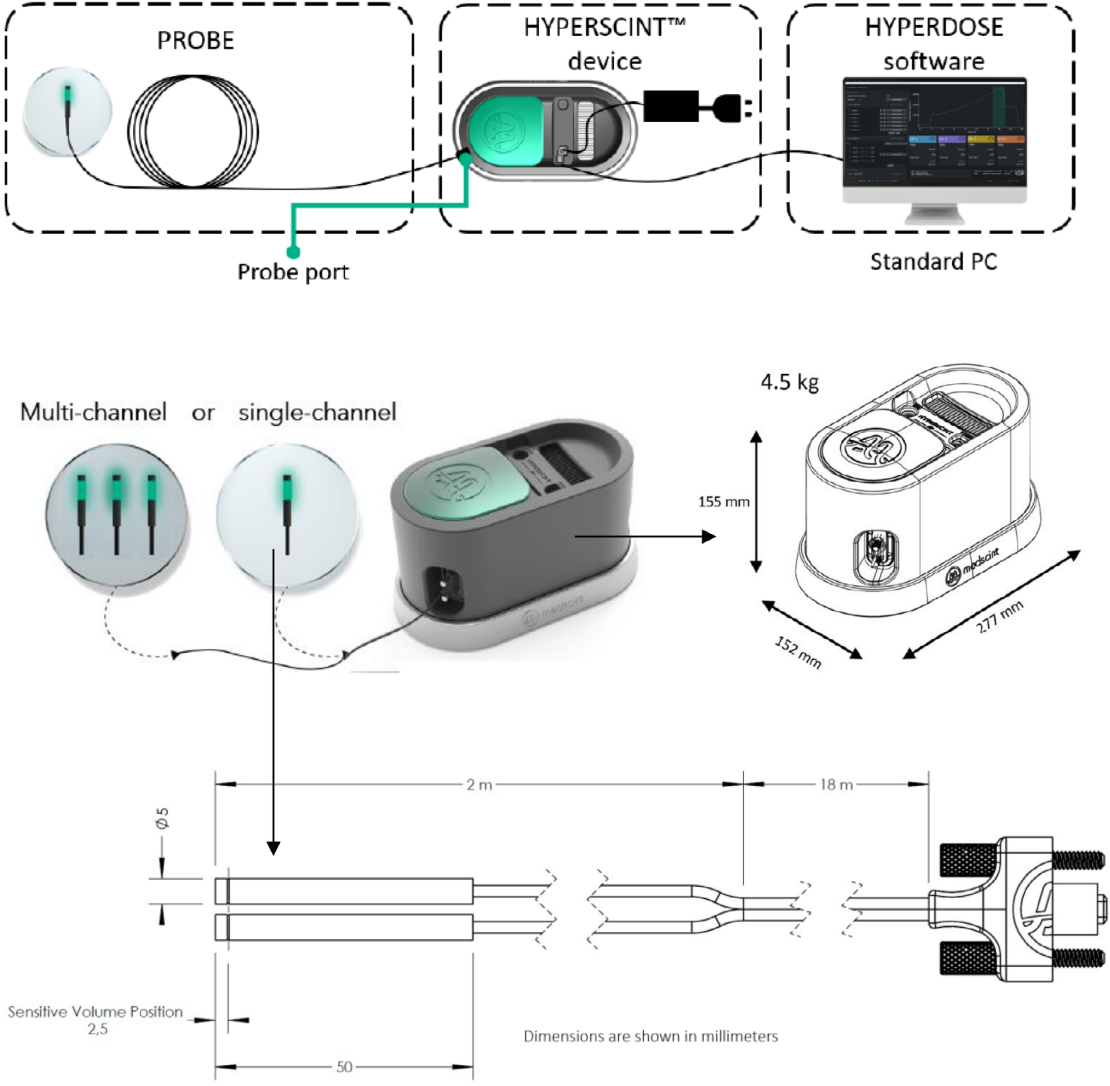
Different components of the Medscint0.5 PSD system including single‐ or multi‐channel probes, Hyperscint reader and Hyperdose software. Probes can come in different formats (single‐channel or multi‐channel) to fit different clinical applications. Figure courtesy of Medscint Inc. Quebec, Quebec, Canada.

All the measurements were performed using the Medscint0.5 PSD system on the Varian TrueBeam. The Medscint0.5 has 3 main components, including single‐ or multi‐channel probes, Hyperscint reader and Hyperdose software (Figure 1). The probe component comes in different designs (single‐channel or multi‐channel) to fit different applications. The Medscint0.5 was calibrated for scintillation, fluorescence, Cerenkov, optical fiber spectral attenuation, and system dose calibration components according to the procedure specified by the manufacturer.^18^ Scintillation and fluorescence calibrations were done using the linac kV imager set to 90 kV, 154 mA, and large focal size. The calibration process accounting for Cerenkov radiation was done in four steps using the highest dose rate of the available MV beam, by placing each channel, above a water phantom tank or on the surface of solid water slabs, 1.5 m from the center of the beam.^18^ This method takes advantage of hyperspectral detection to separate the individual contributions from scintillation, fluorescence, Cerenkov radiation. Each component's spectral signature is characterized during calibration, and a spectral unmixing algorithm is used to assign weights based on their relative contributions. This approach enables implementation of a complete stem effect removal method, allowing for accurate isolation of the scintillation signal by correcting for Cerenkov and other contaminant light sources. This technique is performed in real time during acquisition. These different configurations allow the system to detect Cerenkov radiation and subtract the specific noise signature out to improve SNR, which is essential for small size dosimeters in small field dosimetry. For dose calibration, there are two available calibration methods: individual or grouped probe calibration. In this study, individual probe calibration was performed to avoid uncertainty associated with grouped dose calibration.

The Varian TrueBeam used in this study was equipped with Varian's Stereotactic cones, sized 4, 5, 7.5, 10, 12.5, 15, and 17.5 mm. Irradiations were delivered using a 6 MV‐Flattening Filter Free (FFF) photon beam with dose rates up to 1400 MU/min and a 10 MV‐FFF photon beam with dose rates up to 2400 MU/min. These two beams were favorable in small field stereotactic radiosurgery (SRS) due to their higher dose rates compared to standard flattening filter (WFF) beams (dose rates up to 600 MU/min), especially for cranial functional treatments.^19^, ^20^ Moreover, they provide a wider range of dose rates needed to evaluate the dose rate dependency of the Medscint0.5 detector in this work. The linac is calibrated to deliver 1 cGy per MU in water, at 95 cm SSD at 5 cm depth, with a 10×10 cm^2^ field size (linac calibration condition).

For all measurements, the Medscint0.5 main axis was placed perpendicular to the radiation beam, in the linac calibration condition, unless otherwise noted.

### 2.1 Dose linearity

The dose linearity was evaluated, for both 6 and 10 MV FFF beams, by measuring delivered dose to the Medscint0.5 from 5 up to 2000 MUs, in the linac calibration condition. The linac output linearity was also cross checked using a farmer ion chamber (Model #TN30013, PTW, Freiburg, Germany) in the same setup. The measured signal (dose) was plotted against delivered monitor units to assess dose linearity.

### 2.2 Dose rate

Measured dose was collected from 400 MU/min up to 1400 and 2400 MU/min, respectively, for 6 and 10 MV‐FFF beams. For the linac used in this study, dose rates of < 400 MU/min were not available for both examined beam lines, therefore, 6 MV‐WFF beam was used to evaluate detector dose rate dependency for 10 to 600 MU/min dose rates. For the FFF and WFF beams measurements, 200 and 100 MU were delivered, respectively. Data were normalized to the clinically used dose rates of 1400 and 2400 MU/min, respectively, for 6 and 10 MV‐FFF beams. For the 6 MV WFF measurements, data were normalized to 600 MU/min.

### 2.3 Angular dependence

According to the IAEA TRS‐483, the orientation of the detector axis with respect to the beam central axis has an influence on the measurements for small field dosimetry.^1^ Therefore, it recommends that the central axis of smallest dimension of the detector sensitive volume be aligned to the radiation beam.^1^ In this study, to evaluate angular dependence of the Medscint0.5, measurements were made with the detector central axis positioned parallel and perpendicular to the radiation beam at 95 cm SSD at 5 cm depth for both a 10×10 cm^2^ field size and 4 mm SRS cone. For both detector orientations, the detector was rotated every 90° along its central axis (4 cardinal angles) and measurements were compared to zero degrees. The reading for the perpendicular orientation was compared to the measurement at zero degrees for the parallel position. Moreover, since one drawback of scintillator detector systems is the stem effect due to the collection of Cerenkov light produced in the light guide,^12^, ^13^, ^14^, ^19^ 4 mm cone measurements were conducted to minimize Cerenkov emissions from the detector stem.

### 2.4 Temperature dependence

To assess the temperature dependence of the Medscint0.5, Das et al. methods were utilized.^4^ In this method, the water temperature in a 1D water tank (Sun Nuclear, Melbourne, Florida, USA) was controlled from 16°C to 40°C by adding hot water and later cold ice. To maintain the same amount of attenuating materials along the beam path, the gantry head was rotated to 180 degrees (gantry head up) instead of 0 degrees (gantry head down). Detector depth in the 1D water tank was tuned to obtain an identical reading with 10×10 cm^2^ field size with 0 degrees gantry at 95 cm SSD at 5 cm depth. Readings at different temperatures were normalized to 20.5°C.

### 2.5 Pre‐ and post‐irradiation leakage

Pre‐ and post‐irradiation leakage was determined by measuring dark current signal immediately before and after irradiation. The results were compared with dark current signal measured one minute post irradiation. Poder et al. and Timakova et al. methods were used to determine the Medscint0.5 leakage.^17^, ^20^ According to this method, a 10‐min measurement was done pre‐irradiation. Then, 500 MUs were delivered, followed by a five‐second measurement immediately thereafter. After a 60‐s delay post 500 MUs irradiation, another 5‐s leakage measurement was done. All measurements were performed in the linac calibration condition.

### 2.6 Short‐term repeatability

To determine short‐term repeatability, the Medscint0.5 was irradiated 10 times using 200 MUs every 1 min in the linac calibration conditions. Then, each individual measurement was compared to the average of the readings.

### 2.7 Output factors

The small circular field output factors for Varian SRS cone sizes of 4, 5, 7.5, 10, 12.5, 15, and 17.5 mm and square fields defined by jaws of field sizes of 1.5×1.5, 2×2, 2.5×2.5, 3×3, 5×5, 10×10 cm^2^ were performed. Medscint0.5 specific output factors for each field size were defined as the ratio of the reading at that field size to the reading at a field size of 10×10 cm^2^ (machine specific reference field).^1^, ^21^ Results of the measurements were compared with EGSnrc BEAMnrc MC simulations^22^, ^23^ and PTW microSilicon diode measurements. For the MC simulations, calculated output factors were the ratios of dose to water at central axis. Details of the MC simulations were presented in the previous publications of our team.^22^, ^23^

To find center of the radiation beam, both Medscint0.5 and PTW microSilicon diode detectors were shifted in lateral and superior–inferior directions to find maximum reading, indicating that the active volume of the detectors was perfectly aligned with beam central axis. For microSilicon diode measurements, the field output factors derived from the measurements were daisy‐chained at the 3×3 cm^2^ field size,^1^ using a SNC125 (Sun Nuclear, Melbourne, Florida, USA) ionization chamber. No further correction factors were applied to the results. It is worth mentioning that measurements were not daisy‐chained for Medscint0.5.

Before measuring field output factors, jaw position accuracy versus radiation beam was checked according to the TG‐198 using the EBT3 film method.^24^ No discrepancies were found for X1, X2, and Y1 up to a 10 cm jaw opening. For Y2, less than 0.5 mm discrepancy was investigated.

### 2.8 Uncertainty analysis

In the uncertainty analysis performed in this study, the uncertainty values refer to total uncertainty, calculated by taking a quadrature sum of the Type A and Type B uncertainties.^25^ For all the measurements, Type A uncertainties were derived by calculating standard deviation (1 σ confidence interval) of the measurements.^26^ Each measurement was repeated three times. Type B uncertainties were estimated by performing independent sets of repeated measurements on different days. To assess the uncertainties associated with setup reproducibility and linear accelerator output variations, average standard deviations of the resulting datasets were calculated.

## 3 RESULTS

### 3.1 Dose linearity

**FIGURE 2:**
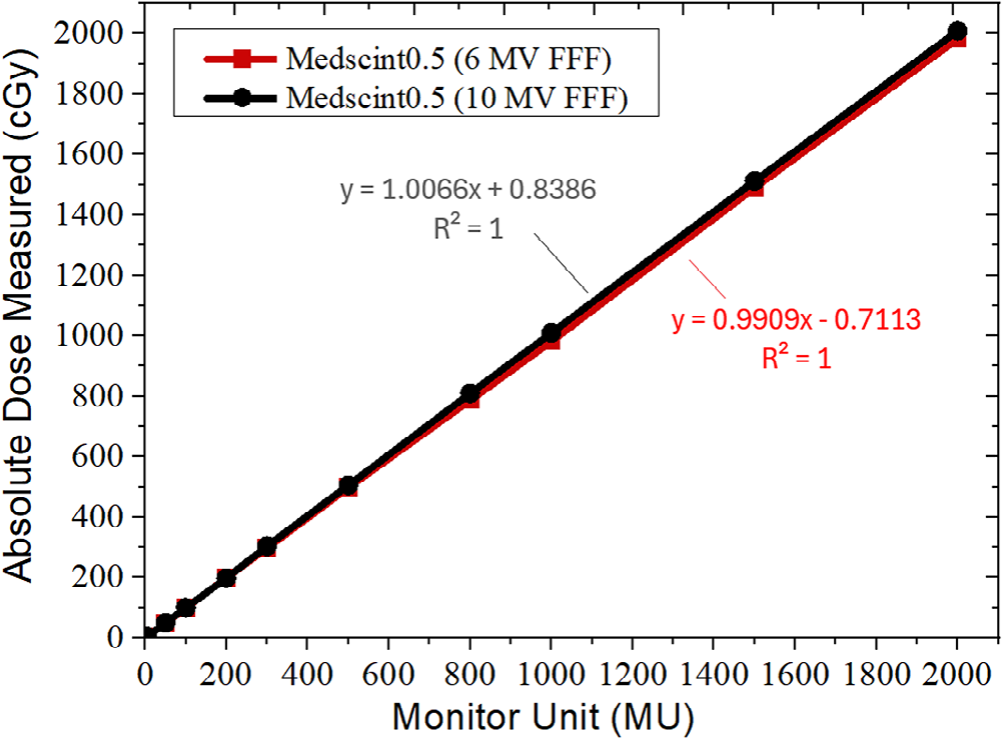
Dose linearity of Medscint0.5 for the measured absolute signal for both 6 and 10 MV FFF beams.

**FIGURE 3:**
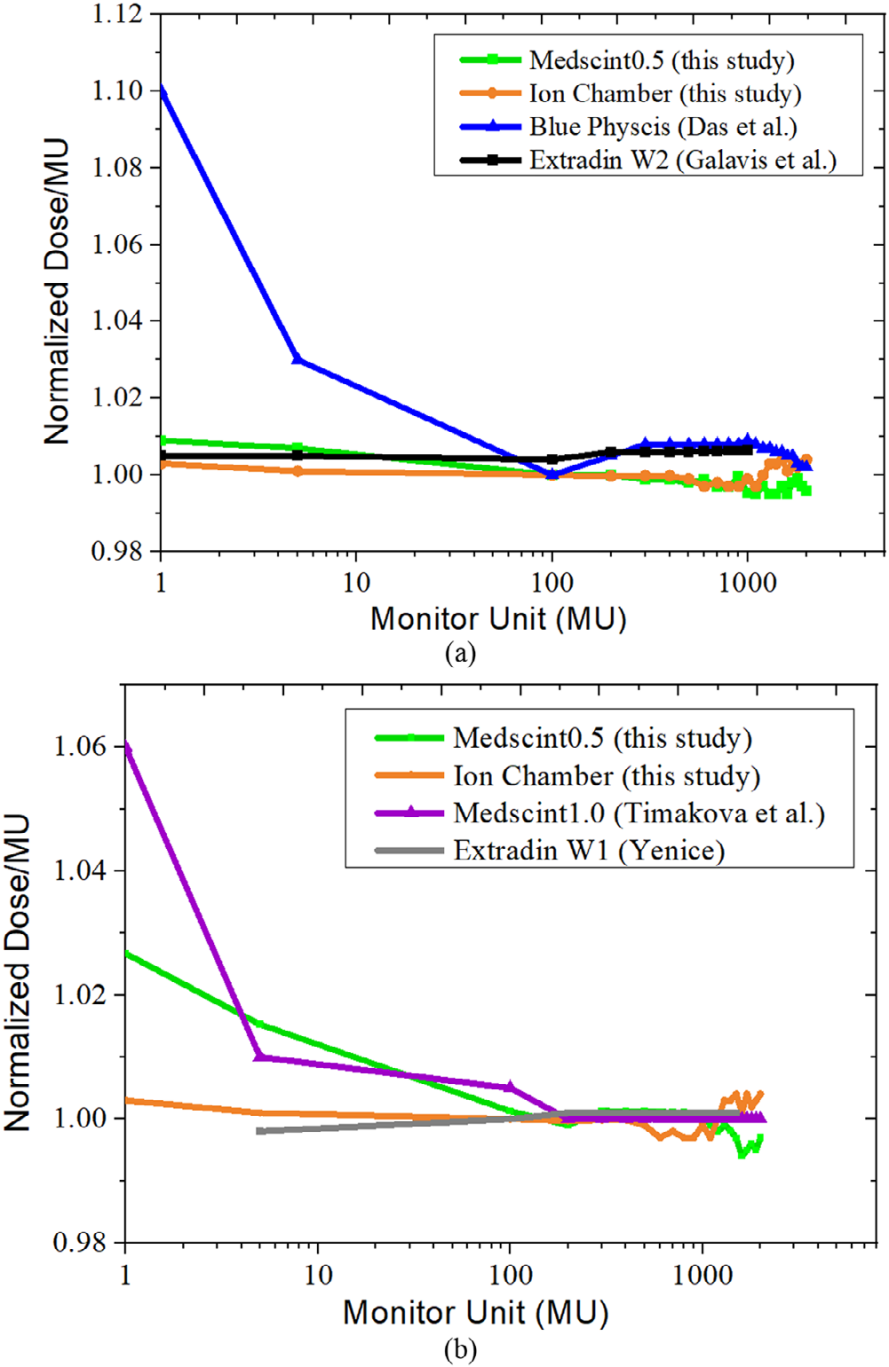
Deviations of normalized measured dose per MU for (a) 6 MV FFF and (b) 10 MV FFF beams.

Figure 2 shows the dose linearity of Medscint0.5 for the measured absolute signal and the deviations of normalized measured dose per MU for both 6 and 10 MV FFF beams. Linac output measurements using the farmer chamber showed less than 0.40% deviation for the range of studied MUs (Figure 3). Linear fit to the Medscint0.5 measured data revealed well defined linearity for both energies (*R* ^2^ ≈ 1.0). For the 6 MV FFF beam, the maximum discrepancy between the measured and nominal dose was < 1.00% for 5 MUs, whereases less than 0.51% difference was seen for MUs between 5 and 2000. Slightly higher discrepancies were observed for the 10 MV FFF beam (up to 0.73%), especially for 5 MUs (up to 2.67%; Figure 3).

### 3.2 Dose rate

**FIGURE 4:**
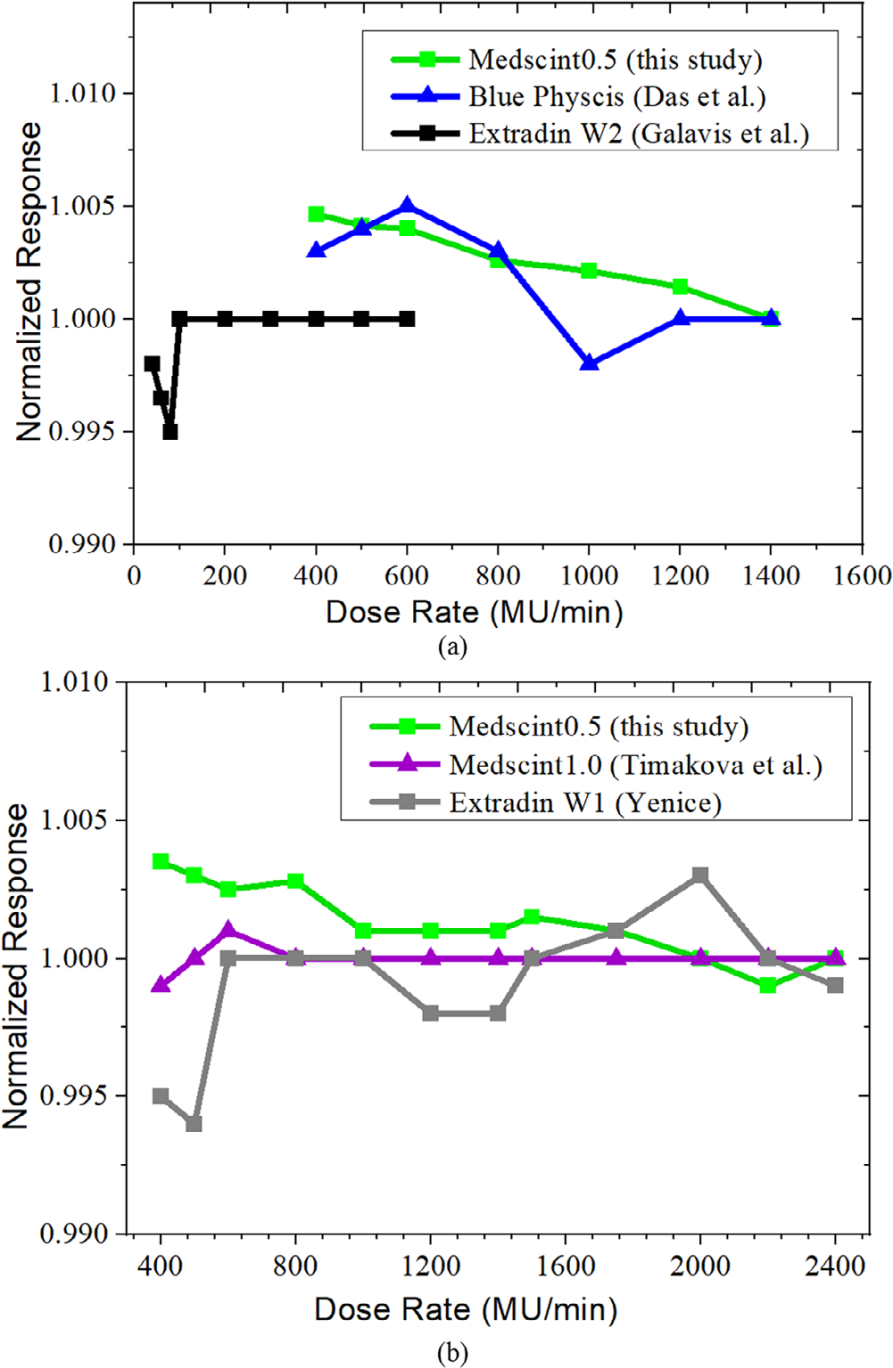
Dose rate response of the Medscint0.5 for (a) 6 MV‐FFF and 6 MV‐WFF and (b) 10 MV‐FFF beams, normalized to clinically used dose rates of 600, 1400, and 2400 MU/min for 6 MV‐WFF, 6 MV‐FFF, and 10 MV‐FFF beams, respectively.

Figure 4 illustrates the dose rate response of the Medscint0.5 normalized to clinically used dose rates of 1400 and 2400 MU/min for 6 and 10 MV FFF beams, respectively, and also 600 MU/min for 6 MV WFF beam. For the 6 MV FFF beamline, the largest discrepancy was observed for a dose rate of 400 MU/min (up to 0.47%). Less than 0.33% differences were seen from 400 to 1400 MU/min. A similar trend was revealed for the 10 MV FFF beam, with the maximum discrepancy for 400 MU/min (up to 0.37%) and < 0.35% for dose rates from 400 to 2400 MU/min (Figure 4). For the 6 MV WFF beam, the largest discrepancy was observed for dose rates of 40–100 MU/min (up to 0.35%). Less than 0.30% differences were seen from 400 to 600 MU/min.

### 3.3 Angular dependence

**FIGURE 5:**
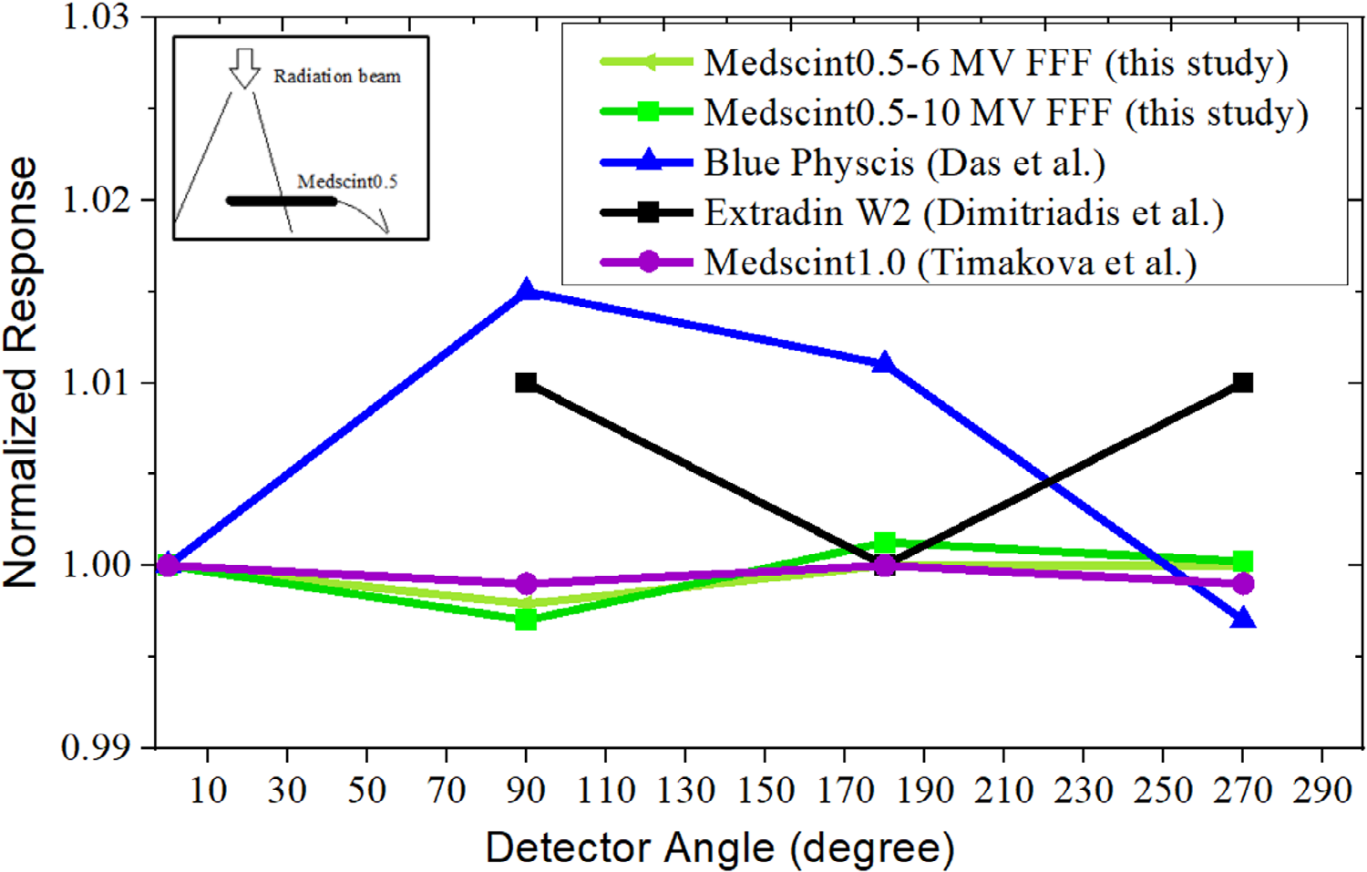
Angular dependency of the Medscint0.5 positioned perpendicular to the 10×10 cm^2^ field radiation beam.

Figure 5 illustrates the response of the Medscint0.5 positioned perpendicular to the 10×10 cm^2^ field radiation beam. For both 6 and 10 MV FFF beams, the maximum angular dependence was < 0.30%. Similar results (angular dependency < 0.30%) were observed for the 4 mm cone measurements, indicating that there was negligible stem effect for this probe.

Conversely, positioning the detector parallel to the radiation beam showed larger variations of 2.95% and 3.91%, respectively, for 6 and 10 MV FFF beams. Compared to zero degree for parallel position, angular dependence was < 0.30% when detector was rotated every 90° along its central axis.

### 3.4 Temperature dependence

**FIGURE 6:**
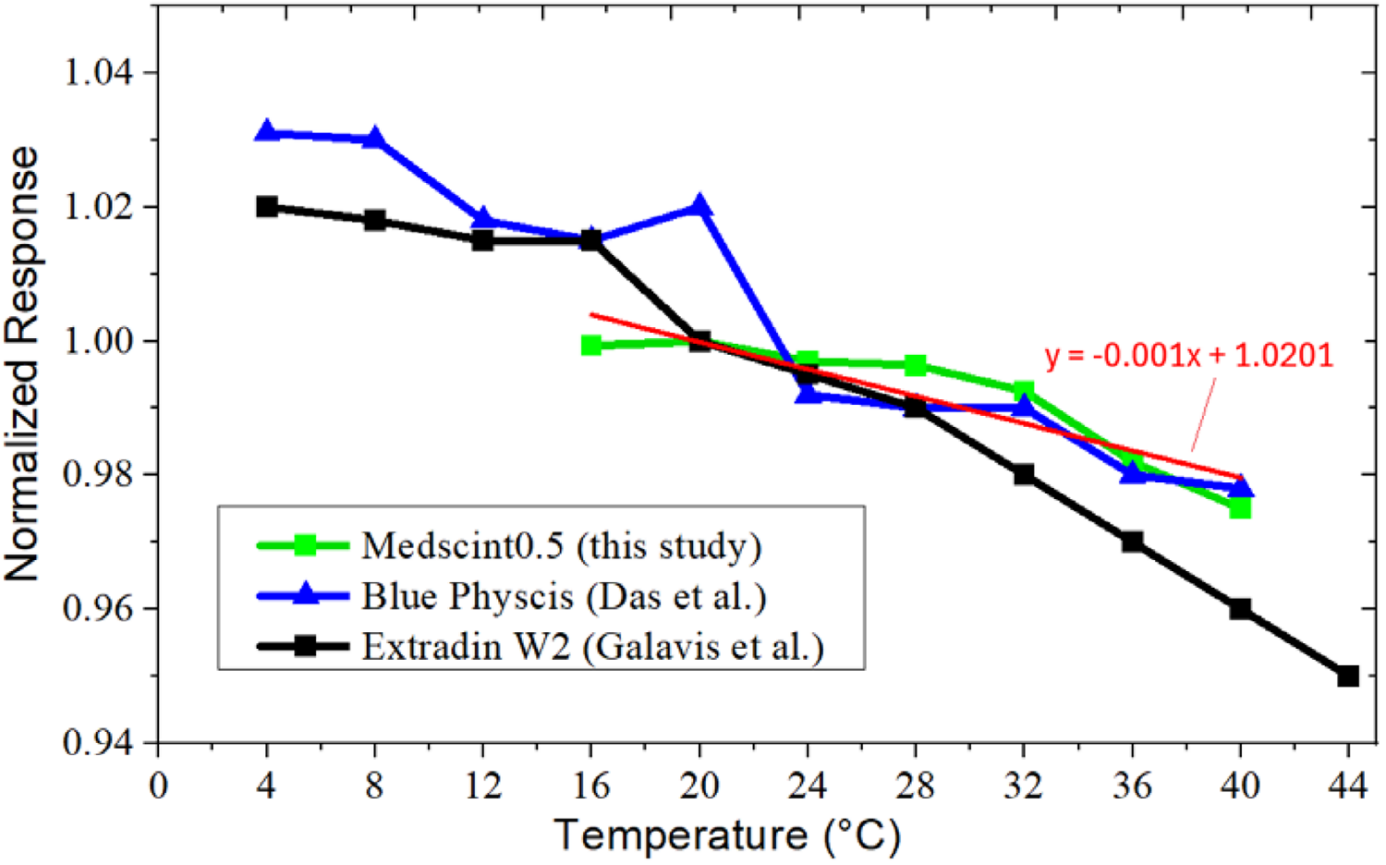
Temperature dependency of the Medscint0.5 for the studied temperatures of 16°C–40°C. Readings at different temperatures were normalized to 20.5°C.

Medscint0.5 showed a slight temperature dependency for the studied temperatures of 16°C–40°C (Figure 6). In this figure, data were normalized to readings at 20.5°C and the Medscint0.5 detector reading revealed a downward trend with increasing temperature. Results showed up to a 2.45% decrease in response at 40°C. For clinically relevant temperatures (∼18°C–24°C), differences < 0.38% were found.

### 3.5 Pre‐ and post‐irradiation leakage

Dark current pre‐irradiation measurements showed a reading of 0.86 cGy. For 6 MV FFF irradiation, the 5‐s immediate post‐irradiation reading was 0.022 cGy and the 5 s reading one minute post irradiation was 0.031 cGy. For 10 MV FFF irradiation, the readings were 0.045 cGy for both immediate and 60 s post irradiation.

### 3.6 Short‐term repeatability

For 6 MV FFF, the average of the measured dose in the linac calibration condition was 199.78 ± 0.09 cGy. Whereas for the 10 MV FFF beam, it was 199.71 ± 0.13 cGy. The maximum difference of the observed reading was 0.07% and 0.09% for 6 and 10 MV FFF beams, respectively.

### 3.7 Output factors

**FIGURE 7:**
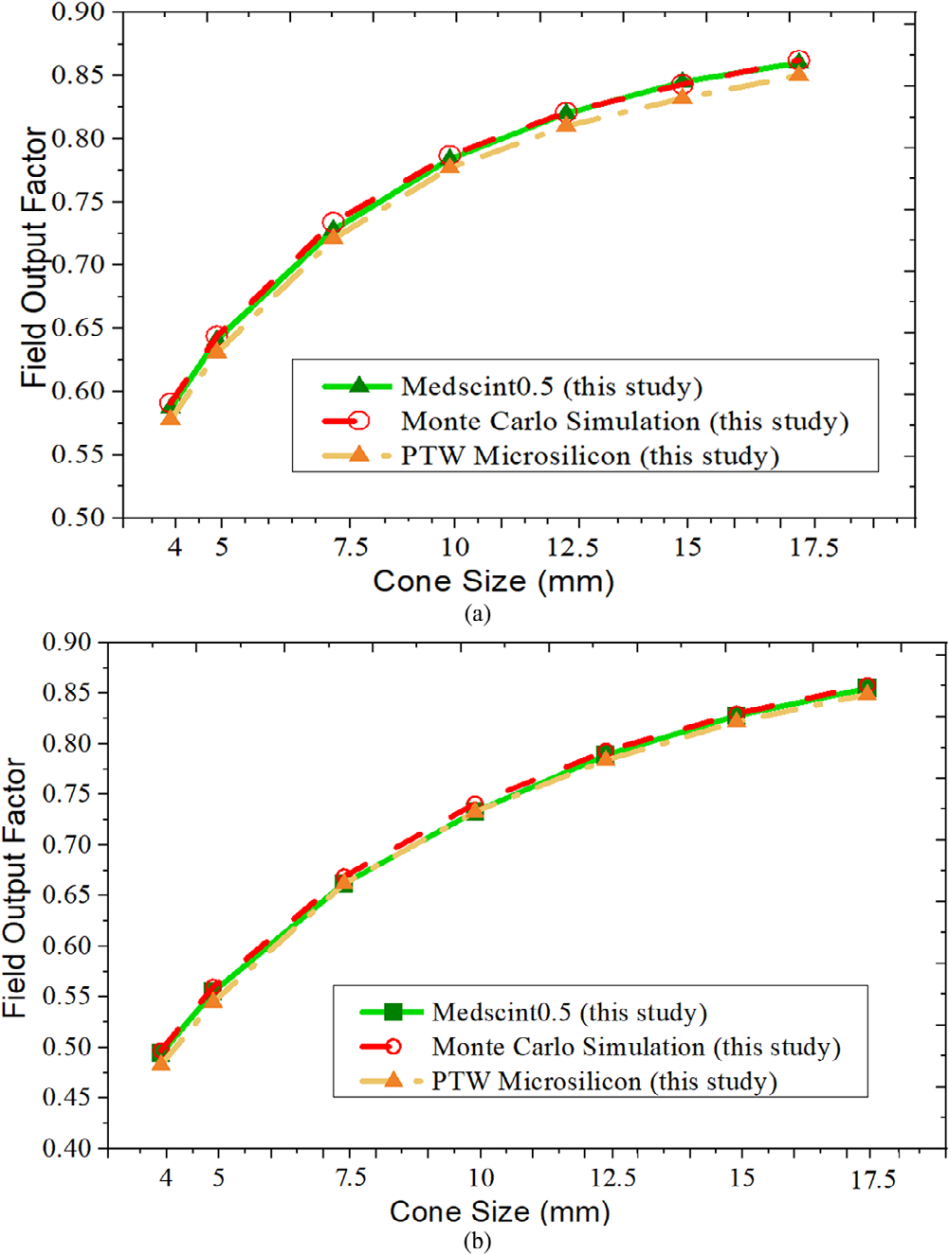
Cone output factors measured by Medscint0.5 and PTW micro‐Silicon diode, and derived data using MC simulations. Results are presented for all the SRS cone sizes for both (a) 6 MV FFF and (b) 10 MV FFF beams.

Figure 7 shows cone output factors measured by Medscint0.5 and PTW micro‐Silicon diode, and derived data using MC simulations. As can be seen from this figure, Medscint0.5 showed agreement within 1.03% with MC for all the SRS cone sizes for both 6 and 10 MV FFF beams. As expected, the maximum discrepancy was found for cone sizes of < 7.5 mm, though differences were less than 0.48% for cone sizes of 10 to 17.5 mm. As shown in Figure 7 and Table 1, average discrepancies of the measured output factors measured by Medscint0.5 for all SRS cone sizes were 0.38% and 0.68% for 6 and 10 MV FFF beams, respectively. For jaw‐defined field sizes, Medscint0.5 results showed an average discrepancy of 0.30% compared to MC for all the energies and studied field sizes.

**TABLE 1:** Comparison of the measured cone and field output factors using Medscint0.5 and PTW micro‐Silicon diode, with MC derived data, for (a) 6 MV FFF and (b) 10 MV FFF beams.

(a)						
		Output factors			|Differences compared to MC (%)|	
Field size (mm)		Medscint0.5	PTW micro‐Silicon diode^a^	MC	Medscint0.5	PTW micro‐Silicon diode
Cone	4.0	0.587	0.578	0.591	0.76	2.20
	5.0	0.641	0.631	0.644	0.47	2.02
	7.5	0.729	0.721	0.734	0.68	1.77
	10.0	0.784	0.772	0.787	0.38	1.91
	12.5	0.820	0.810	0.821	0.15	1.34
	15.0	0.845	0.833	0.843	0.18	1.19
	17.5	0.860	0.851	0.862	0.23	1.28
Jaw	15 × 15 mm^2^	0.838		0.839	0.12	
	20 × 20 mm^2^	0.867		0.869	0.23	
	25 × 25 mm^2^	0.884		0.889	0.56	
	30 × 30 mm^2^	0.899		0.904	0.55	
	50 × 50 mm^2^	0.943		0.943	0.03	

(b)						
		Output factors			|Differences compared to MC (%)|	
Field size (mm)		Medscint0.5	PTW micro‐Silicon diode^a^	MC	Medscint0.5	PTW micro‐Silicon diode^a^
Cone	4.0	0.494	0.483	0.497	0.51	2.82
	5.0	0.555	0.545	0.560	0.85	2.68
	7.5	0.662	0.662	0.669	1.02	1.05
	10.0	0.733	0.733	0.741	1.03	1.08
	12.5	0.789	0.784	0.793	0.48	1.13
	15.0	0.828	0.822	0.830	0.25	0.96
	17.5	0.855	0.849	0.858	0.36	1.05
Jaw	15 × 15 mm^2^	0.834		0.828	0.52	
	20 × 20 mm^2^	0.881		0.882	0.07	
	25 × 25 mm^2^	0.908		0.910	0.24	
	30 × 30 mm^2^	0.925		0.930	0.48	
	50 × 50 mm^2^	0.963		0.963	0.03	

^a^ PTW microSilicon diode measurements were daisy‐chained at the 30×30 mm^2^ field size, using a SNC125 ionization chamber. No further correction factors were applied to the results.

Daisy‐chained measurements of PTW micro‐Silicon diode also demonstrated good agreement with MC simulations (< 2.82%) for all the SRS cone sizes (Figure 7). Better agreements (< 1.34%) were seen for cone sizes of 12.5–17.5 mm. Like Medscint0.5 measurements, results for 6 MV FFF beam showed better agreement with MC compared to the 10 MV FFF beam (Table 1). For all SRS cone sizes, average discrepancies of the measured output factors using the PTW diode were 1.54% and 1.67% for 6 and 10 MV FFF beams, respectively (Figure 7 and Table 1).

### 3.8 Uncertainty analysis

Results of the uncertainty analysis are presented in Table 2. This table shows the total combined uncertainty associated with using Medscint0.5 for small field dosimetry using SRS cones.

**TABLE 2:** Uncertainty analysis of using Medscint0.5 for absolute small field dosimetry using SRS cones.

	Standard uncertainty (%)	
Source	Type A	Type B
Cross calibration using an ion chamber		±0.7%
Medscint0.5 calibration for scintillation, fluorescence, Cerenkov, optical fiber spectral attenuation, and system dose calibration		±0.3%
Radiation leakage	±0.1%	
Short‐term repeatability	±0.1%	
Angular dependency	±0.3%	
Temperature dependency for 18°C–24°C	±0.4%	
Reproducibility in output measurements	±0.1%	
Setup accuracy		±0.5%
Combined	±0.53%	±0.91%
Total uncertainty	±1.05%	

## 4 DISCUSSION

This work studied dosimetric characteristics of the Medscint0.5 PSD for small field dosimetry. Results from this study were compared with published data for other commercially available detector systems, including other PSDs. Timakova et al. has previously characterized a previous model, called Medscint1.0, for 6 MV with flattening filter (WFF) and 10 MV FFF beams.^17^ In addition to the smaller size advantage, which is beneficial for small field dosimetry due to increased spatial resolution and decreased volume averaging, particularly SRS cone sizes down to 4 mm, the added feature to this model is the more advanced signal processing, spectral binning, and an upgraded five‐component calibration.^18^

Results of dose linearity evaluations showed less than 1% and 2.7%, respectively, for 6 and 10 MV FFF, deviation between the delivered MU and measured dose (Figure 2). This is in agreement with the Medscint1.0 detector (nonlinearity < 1.5%) for MUs ≥ 5, and marginally better than Blue Physics (Figure 3) for which Das et al.^4^ have reported a slight nonlinearity of more than 3% for MUs < 5. Galavis et al.^8^ have characterized Exradin W2 for small field dosimetry and reported less than 0.5% dose non‐linearity even for small MUs. It should be noted that Galavis et al. have reported no chamber measurements to assess beam linearity for comparison purposes. According to the previously published reports, dose linearity is mainly related to accuracy of Cerenkov subtraction from the main signal.^12^, ^14^, ^27^ Therefore, independent of radiation energy, Medscint0.5 can accurately provide linear dose response based on the processed SNRs for both low and high doses.

Scintillation detectors have been shown to provide linear response even in extreme ion recombination with high dose rates (> 1400 MU/min). Our findings for Medscint0.5 showed < 0.5% deviations of the dose response for dose rates of 400–2400 MU/min (Figure 4). The linac used in this study did not have dose rates of < 400 MU/min for the studied beamline. However, most centers now use FFF beams with dose rates of > 1000 MU/min for SRS delivery. Das et al. have tested the Blue Physics for both 6 MV WFF and 6 MV FFF beams and reported < 0.5% differences for dose rates > 400 MU/min.^4^ Blue Physics has shown no visible signal saturation in high dose rate regions and < 1% differences at low dose rate regions of 100 MU/min.^4^ Timakova et al. have shown that the Medscint1.0 has a linear response (< 0.5%) for dose rates between 200 and 2400 MU/min, although differences increased upwards 1.56% for lower dose rates of 40 MU/min.^17^ Similar observations (< 1.0%) were revealed for the Exradin W1 Scintillator detector characterized by Kamil Yenice^28^ (Figure 4) as well, indicating the Medscint0.5 is running right in line with other comparable devices. It should be noted that, dose rate dependency between different PSD systems might be related to differences in signal processing and spectral binning.

Medscint0.5 showed less than 0.3% angular dependency for both 6 and 10 MV FFF beams, when positioned perpendicular to the radiation beam (Figure 5). This finding is in line with Medscint1.0, for which < 0.2% dependency has been reported.^17^ It is worth mentioning that for Medscint1.0, the detector has been placed in the center of a cylindrical phantom and irradiated at different gantry angles.^17^ While in our study, to minimize uncertainties related to gantry rotation and sag,^29^, ^30^ the detector was rotated every 90° along its central axis. Moreover, to minimize uncertainty related to Cerenkov emissions from the stem and cables, we repeated the experiment using the 4 mm cone to expose just the sensitive volume of the detector. No significant differences were seen for angular dependency measurements using the 4 mm cone and linac calibration field size (< 0.3% differences). The Blue Physics detector has shown to have slightly higher angular dependency (up to 1.5%) (Figure 5), which might be related to its construction housing materials.^4^, ^31^ Dimitriadis et al. have reported < 0.3% and < 1.0% angular dependencies when the Extradin W2 was positioned perpendicular and parallel, respectively, to the radiation beam.^26^ When positioning the Medscint0.5 parallel to the radiation beam, we observed up to 3.9% differences compared to zero degree for the parallel position, indicating the importance of keeping the detector in the same orientation while collecting a set of data for reproducibility used, especially for small filed dosimetry.^1^, ^2^

Since dosimetry using Medscint0.5 might be performed in patients/phantoms with variable temperatures (up to 40°C), temperature dependency of this detector, as a solid‐state detector, was evaluated from 16 to 40°C (Figure 6). Results showed a similar downward trend compared to the Blue Physics^4^ detector (< 1.0% differences) and lower (up to 2%) than Exradin W2^8^ detector for temperature of 40°C (Figure 6). Though for Blue Physics, Das et al. conducted experiments for temperatures down to 4°C and reported up to 3% differences compared to 21.6°C measurements.^4^ Galavis et al.^8^ have reported lower differences (< 1%) than Blue Physics at 4°C, showing that the Exradin W2 detector might be more stable at very low temperatures (< 8°C). Since temperatures of 18 to 24°C are widely relevant in most phantom dosimetry experiments, in our study we did not conduct measurements for temperatures < 16°C. For this range, all the detectors compared in this study illustrated negligible variations in response (less than 0.4%) (Figure 6), which shows temperature independence of the intensity and spectral distribution of the Cerenkov light of the main signal.

Irradiation leakage and short‐term repeatability evaluations revealed that, although this detector is stable for reproducibility (< 0.1%), it might show up to 0.86 cGy reading when collecting dark current for 10 min. This part of the study was repeated at different ambient light settings and similar results were revealed (< 0.1% differences). This is in a good agreement with Timakova et al.^17^ evaluations for Medscint1.0, where up to 0.91 cGy was recorded for a similar setting. The higher value observed before irradiation can be considered normal and is likely due to random electronic noise or minor signal fluctuations. Over time, this noise averages out to around zero and therefore should not significantly impact the measurements. The post‐irradiation values are consistent with the expected behavior of dark current gradually returning to baseline. To our knowledge, no detailed investigations have been reported for Blue Physics leakage. It should be noted that using FFF beams with dose rates of 1400–2400 MU/min, the 0.86 cGy corresponds to < 0.1% of the delivered dose.

In this work, given the uncertainties associated with positioning accuracy and reproducibility of Multileaf Collimators (up to ± 0.5 mm)^32^, ^33^ all small field output factors were measured using Varian SRS cones. Our results showed that measured output using Medscint0.5 was in good agreement with MC derived data for both 6 and 10 MV FFF beams, with less than 0.76% and 1.2% differences, respectively (Figure 7 and Table 1), even at the smallest 4 mm cone sizes. Therefore, this detector needs no correction factors for acceptably accurate small field dosimetry measurements. We repeated output factor measurements for small and intermediate squared field sizes of 1.5×1.5 cm^2^ to 5×5 cm^2^, and similar results (< 0.56% for any FFF energies) were observed, showing the consistent accuracy of this dosimeter for any radiation field size with energies of 6 and 10 MV FFF. In line with our work, it has been shown that no correction factors are needed for Blue Physics and Exradin W2 when used in small field dosimetry. For comparison purposes, PTW micro‐Silicon diode was also used for only SRS cone measurements (Figure 7). Some studies have reported that this detector can be a correction free detector in small field dosimetry, when daisy‐chained, with some exceptions.^7^, ^34^, ^35^ For the PTW micro‐silicon measurements, 2.82% was the best observed agreement compared to the MC derived data for the 4 mm SRS cone. Slightly better agreement (2.2%) was seen for 6 MV FFF beams, yet the average discrepancies of the measured output factors using PTW micro‐Silicon diode was < 1.67% (Figure 7). Results of our PTW micro‐silicon measurements were in good agreement with Weber et al. who reported up to 3.1% differences with MC for the smallest 5 mm collimator of Cyberknife.^7^

Table 2 illustrates the uncertainty analysis of using Medscint0.5 for small field dosimetry. According to this table, the total uncertainty for absolute dosimetry using this detector is about 1.1% at one standard deviation, mainly due to 0.7% uncertainty associated with cross calibration using an Accredited Dosimetry Calibration Laboratory (ADCL) calibrated ion chamber.^36^ The uncertainty can be reduced to 0.8% at one standard deviation if this detector is used for relative small field dosimetry, that is, field output measurements. On the other hand, with the number of simulated histories used, the uncertainty associated with statistical (quantum) uncertainty in the MC‐calculated results was < 0.10% at the location of the detector in the linac calibration condition. The total uncertainties associated with derivation of the SRS cone output factors were estimated to be < 0.5% when other uncertainties including ionization cross sections, linac geometry, and source spectrum were taken into consideration.

One feature that is under development for this new detector is to use this detector for measuring dose profiles. The current Medscint0.5 dosimetry system is an independent detector that does not wholly integrate with major commercially available 3D dosimetry scanning systems’ ability to record both dose response and spatial information simultaneously. While there are in‐house solutions currently under development to address this issue, profiles and PDDs were not accounted for until robust validation on that methodology has been shown. Moreover, this study evaluated this detector's characteristics for small field dosimetry using only 6 and 10 MV FFF with dose rates > 400 MU/min. Full validation of the Medscint0.5 detector for general dosimetry in clinical practice needs inclusion of other beam spectrums and dose rates < 100 MU/min. In addition, larger field size evaluations (> 10×10 cm^2^) and possibly multicentric studies of this detector, may provide more comprehensive data with higher capability on the characteristics of Medscint0.5 for general clinical dosimetry.

## 5 CONCLUSION

We characterized the performance of the HYPERSCINT Medscint 0.5 ×0.5 mm plastic scintillating detector in experimental measurements and found that it is well suited for small field dosimetry. One of the most important findings is that this latest small size detector requires no corrections in measuring small field output factors even at the smallest circular field of 4 mm diameter.

## AUTHOR CONTRIBUTIONS

All authors participated in data collection, analysis, writing, and editing of the manuscript.

## CONFLICT OF INTEREST STATEMENT

The authors declare no conflicts of interest.
